# Supplementary material for: Viral sequence determines HLA-E-restricted T cell recognition of hepatitis B surface antigen
Source: Nat Commun. 2024 Nov 22;15:10126. doi: 10.1038/s41467-024-54378-9 (PMC11584656; doi:10.1038/s41467-024-54378-9)
Supplement: Supplementary file 1 — Supplementary Information [file 41467_2024_54378_MOESM1_ESM.pdf]

# Viral sequence determines HLA-E-restricted T cell recognition of hepatitis B surface antigen

Gavuthami Murugesan[1ϕ], Rachel L. Paterson[1ϕ], Rakesh Kulkarni[1], Veronica Ilkow[1], Richard J. Suckling[1], Mary M. Connolly[1], Vijaykumar Karuppiah[1], Robert Pengelly[1], Archana Jadhav[1], Jose Donoso[1], Tiaan Heunis[1], Wilawan Bunjobpol[1], Gwilym Philips[1], Kafayat Ololade[1], Daniel Kay[1], Anshuk Sarkar[1], Claire Barber[1], Ritu Raj[1], Carole Perot[1], Tressan Grant[1], Agatha Treveil[1], Andrew Walker[1], Marcin Dembek[1], Dawn Gibbs-Howe[1], Miriam Hock[1], Ricardo J. Carreira[1], Kate E. Atkin[1], Lucy Dorrell[1], Andrew Knox[1], Sarah Leonard[1], Mariolina Salio[1], Luis F. Godinho[1]*.

[1]Immunocore Ltd, 92 Park Drive, Abingdon, Oxfordshire, OX14 4RY, UK.

[ϕ]These authors contributed equally: Gavuthami Murugesan, Rachel L. Paterson.

Correspondence should be addressed to L.F.G. (email: luis.godinho@immunocore.com)

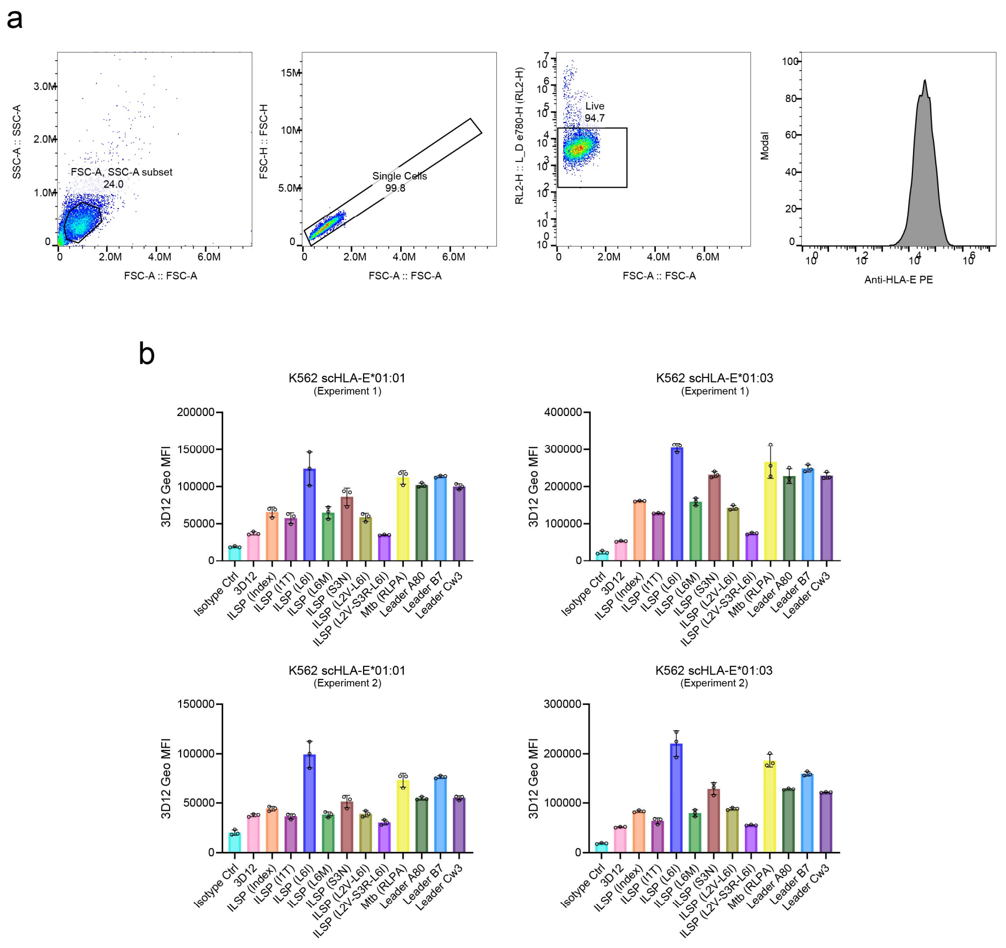


**Supplementary Figure 1. Flow cytometry measurement of HLA-E surface expression in K562-E*01:01 and K562-E*01:03.** HBV Env$_{371\text{-}379}$ variants, Mtb RLPA and signal peptides (A80, B7 and Cw3) binding to HLA-E (01:01, left panels; 01:03, right panels) was measured by scHLA-E up-regulation at the cell surface by flow cytometry using 3D12 antibody (HLA-E antibody). Cells were pulsed with 100 µg/ml for 16 h before analysis. **a** Gating strategy used to analyse HLA-E expression on singlets and live K562-E*01:01 or K562-E*01:03 cells presented on Fig. 1b. **b** Mean fluorescence intensity (MFI) of HLA-E (3D12) plotted. Data are represented as mean ± SD of two independent experiments. Source data are provided as a Source Data file.

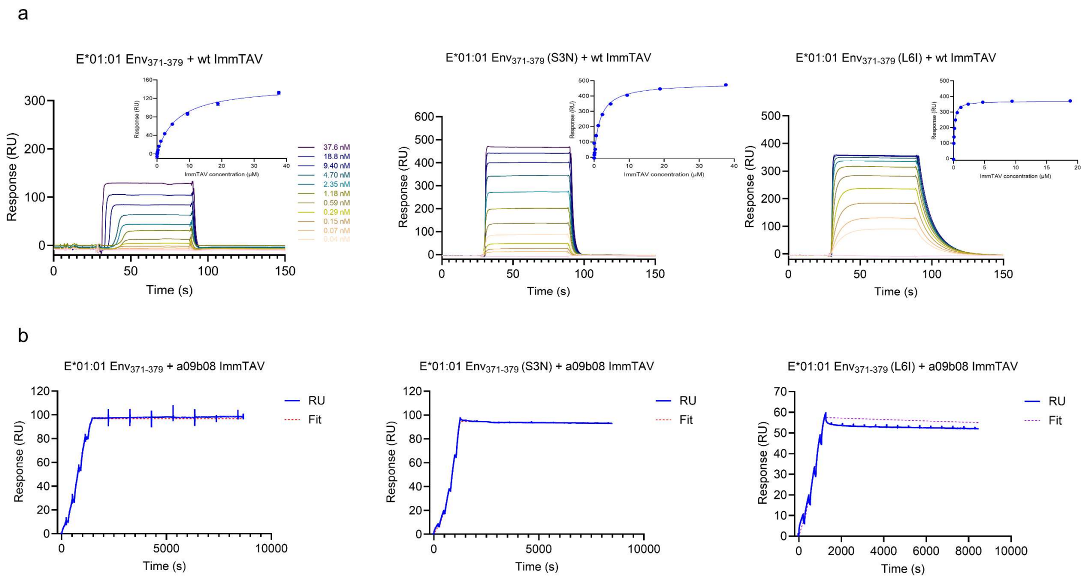


**Supplementary Figure 2. Binding kinetics of wild type and a09b08 ImmTAVs to HBV pHLA-E*01:01 complexes. a** Binding curves for WT ImmTAV and all three HBV pHLA-E*01:01 complexes. Binding was determined over a range of analyte concentrations from 37 nM to 37.6 µM. Insets: calculation of steady-state affinity, data are presented as mean values ± SD. **b** Binding kinetics for a09b08 ImmTAV and all three HBV pHLA-E*01:01 complexes. Graphs show the mean of the raw data (blue) and the 1:1 fit (dotted red line). For the binding kinetic graphs, ImmTAV was flown over the chip as the analyte at concentrations ranging from 0.313 to 5 nM. Kinetic constants were determined using a 1:1 Langmuir model. All experiments were performed at 25°C in triplicate. Data are presented as mean values ± SD of triplicates. Source data are provided as a Source Data file.

a

| pHLA-A*02:01 | pHLA loaded (RU) | Folded pHLA (%) | a09b08 binding (RU) | pHLA ($t_{1/2}$ h) | pHLA (Tm °C) |
|---|---|---|---|---|---|
| $Env_{371-379}$ | 95 | 30.7 | 0 | 17.2±7.7 | 63.9±0.1 |
| $Env_{371-379}$ (S3N) | 110 | 26.5 | 0.5 | 16.8±7.5 | 63.6±0.1 |
| $Env_{371-379}$ (L6I) | 98 | 29.8 | 1.1 | 19.0±7.4 | 65.1±0.1 |

b

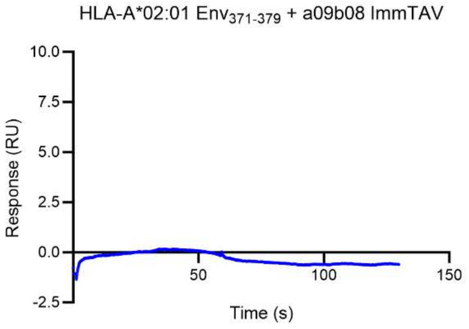


c

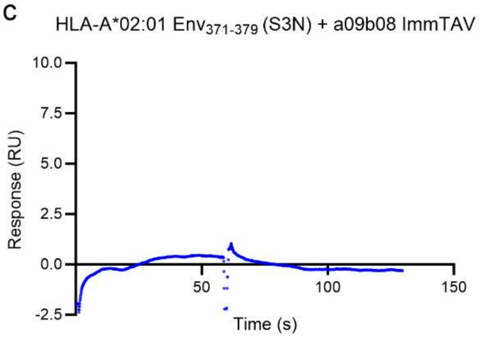


d

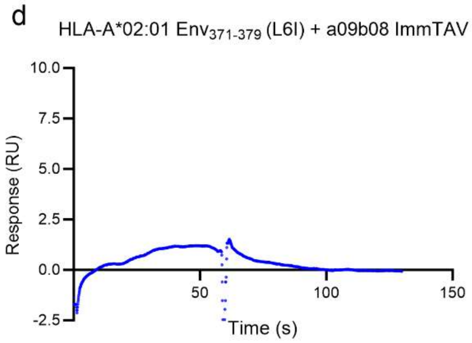


**Supplementary Figure 3. The a09b08 ImmTAV does not bind to pHLA-A*02:01 complexes loaded with HBV $Env_{371-379}$ peptides. a** Summary table of the SPR experimental conditions. **b** Sensorgram for a09b08 ImmTAV (10 nM) and HBV $Env_{371-379}$ pHLA-A*02:01 complexes. **c** Sensorgram for a09b08 ImmTAV (10 nM) and HBV $Env_{371-379}$ (S3N) pHLA-A*02:01 complexes. **d** Sensorgram for a09b08 ImmTAV (10 nM) and HBV $Env_{371-379}$ (L6I) pHLA-A*02:01 complexes. All experiments were performed at 25°C. Data are represented as mean ± SD. Source data are provided as a Source Data file.

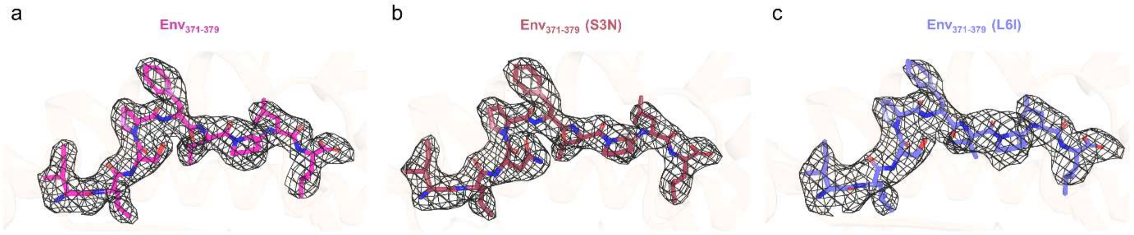


**Supplementary Figure 4. Illustration of the electron density maps for the HBV peptides within the TCR-pHLA-E complexes.** Fo-Fc omit Fourier electron density maps of $Env_{371-379}$ in magenta (**a**) $Env_{371-379}$ (S3N) in red (**b**) and $Env_{371-379}$ (L6I) in blue (**c**) peptides in the TCR-pHLA-E complexes. The electron density (black mesh) was contoured at 3σ.

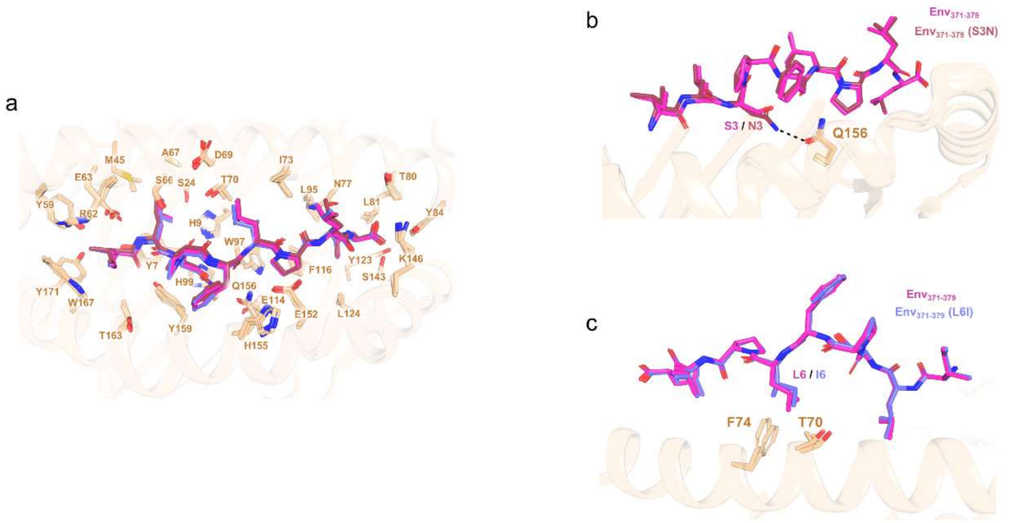


**Supplementary Figure 5. Conformation of the three peptide variants in the HLA-E*01:03 binding groove.** **a** Overlay of the three TCR-pHLA-E complexes aligned on HLA-E. The Env$_{371-379}$ peptide is coloured in magenta, Env$_{371-379}$ (S3N) in red and Env$_{371-379}$ (L6I) in blue. HLA-E residues within 4 Å of the peptides are displayed as sticks. TCR chains are omitted for clarity. **b** Overlay as in **a** showing distance between N3 in the Env$_{371-379}$ (S3N) peptide and Q156 in HLA-E is shorter than with S3 in the Env$_{371-379}$ peptide. The dotted line indicates polar contact. **c** Overlay as in **a** showing conformational difference between the Env$_{371-379}$ and Env$_{371-379}$ (L6I) variants at peptide position 3 and surrounding residues in HLA-E.

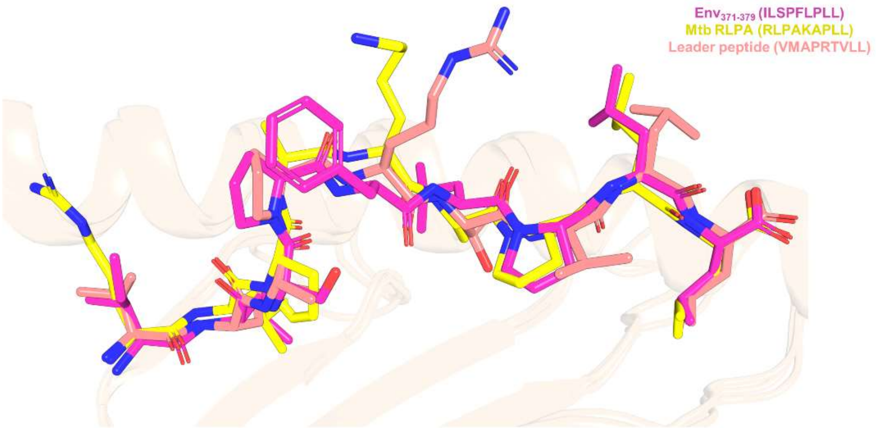


**Supplementary Figure 6. Profile view of superimposition via the HLA peptide-binding grooves for three peptides in HLA-E*01:03.** Comparison of Env$_{371-179}$ (ILSPFLPLL, in purple), Mtb RLPA (in yellow, PDB 6GH1) and HLA-B7 signal sequence (in salmon, PDB 1MHE) peptides alignment on HLA-E*01:03. The MHC α1 helix is shown in wheat.

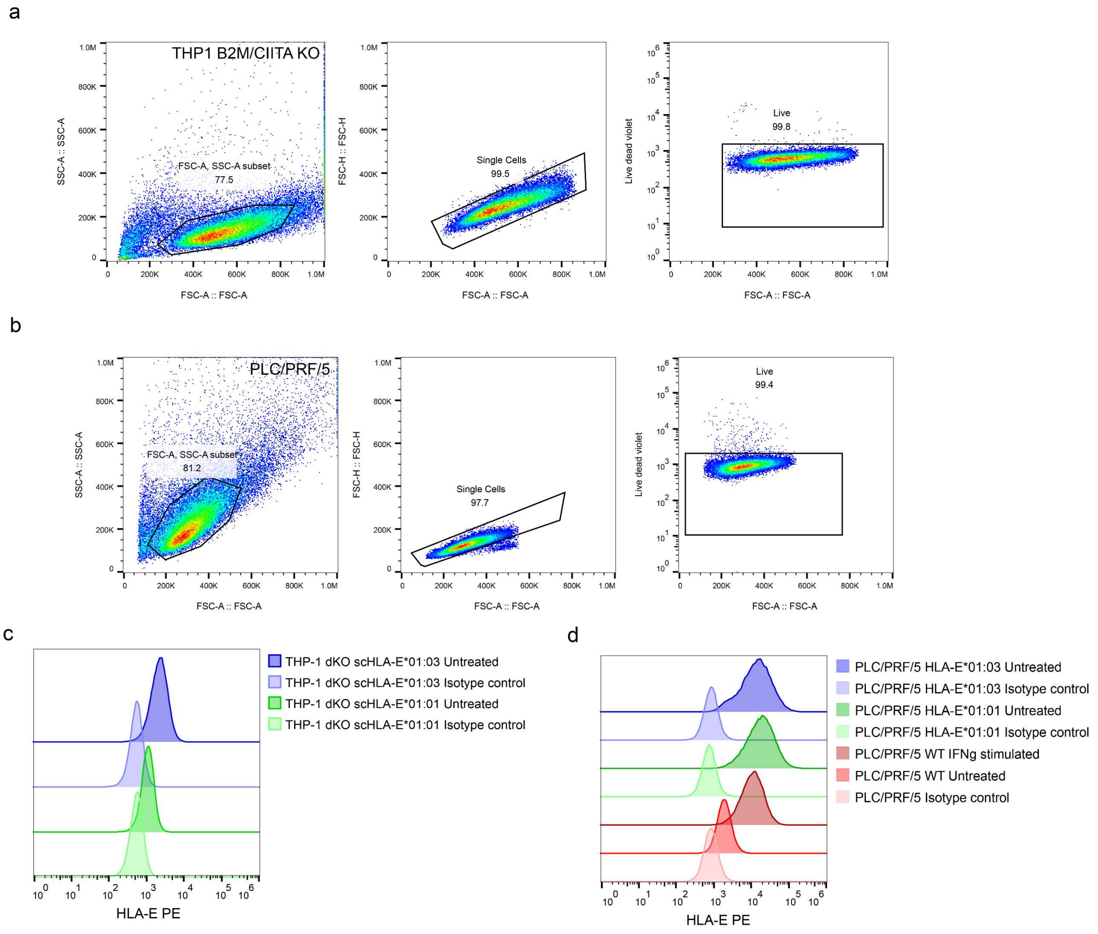


**Supplementary Figure 7. Flow cytometry measurement of cell surface HLA-E expression.** THP1 cells knock-out for *CIITA* and *B2M* (dKO, lacking endogenous HLA class I and II) (**a, c**) and PLC/PRF/5 (**b, d**) were transduced to overexpress single chain HLA-E*01:01 or 01:03 or treated overnight with 1 ng/mL of INF-γ. Cell surface HLA-E was detected by flow cytometry after staining with the anti-HLA-E (3D12) antibody. Gating strategy used to analyse singlets and live THP1 dKO (**a**) and PLC/PRF/5 (**b**) cells. Histograms (**c**, **d**) show HLA-E expression and are representative of three independent experiments.

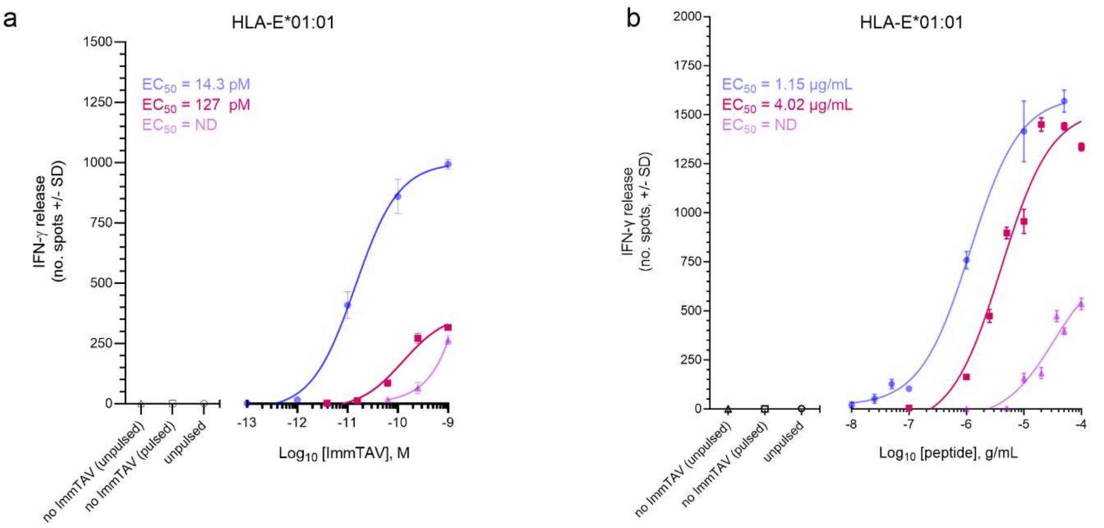


**Supplementary Figure 8. The a09b08 ImmTAV molecule elicits T cell responses against cells displaying HBV pHLA-E complexes. a** ELISPOT assay measuring dose-dependent IFN-γ release induced by a09b08 ImmTAV in co-cultures of PBMC from 3 HBV-naïve donors and THP-1-E*01:01 cells pulsed with $Env_{371-379}$, $Env_{371-379}$ (S3N) and $Env_{371-379}$ (L6I) peptides (10 µg/mL). **b** IFN-γ ELISPOT assays showing titratable activation of PBMC from 3 HBV-naïve donors by a09b08 ImmTAV (1 nM) in the presence of THP-1-E*01:01 cells pulsed with the indicated amounts of peptide. Controls **(a-b**, clear symbols**)** include PBMC + target cells (no ImmTAV; unpulsed or pulsed with 10 µg/mL peptide), and PBMC + ImmTAV + target cells (unpulsed). Average $EC_{50}$ are indicated in the inset at the top left corner of the figures. Data plotted as mean ± SD of triplicates. All donor $EC_{50}$ values and averages are displayed in Supplementary Table 5. Source data are provided as a Source Data file.

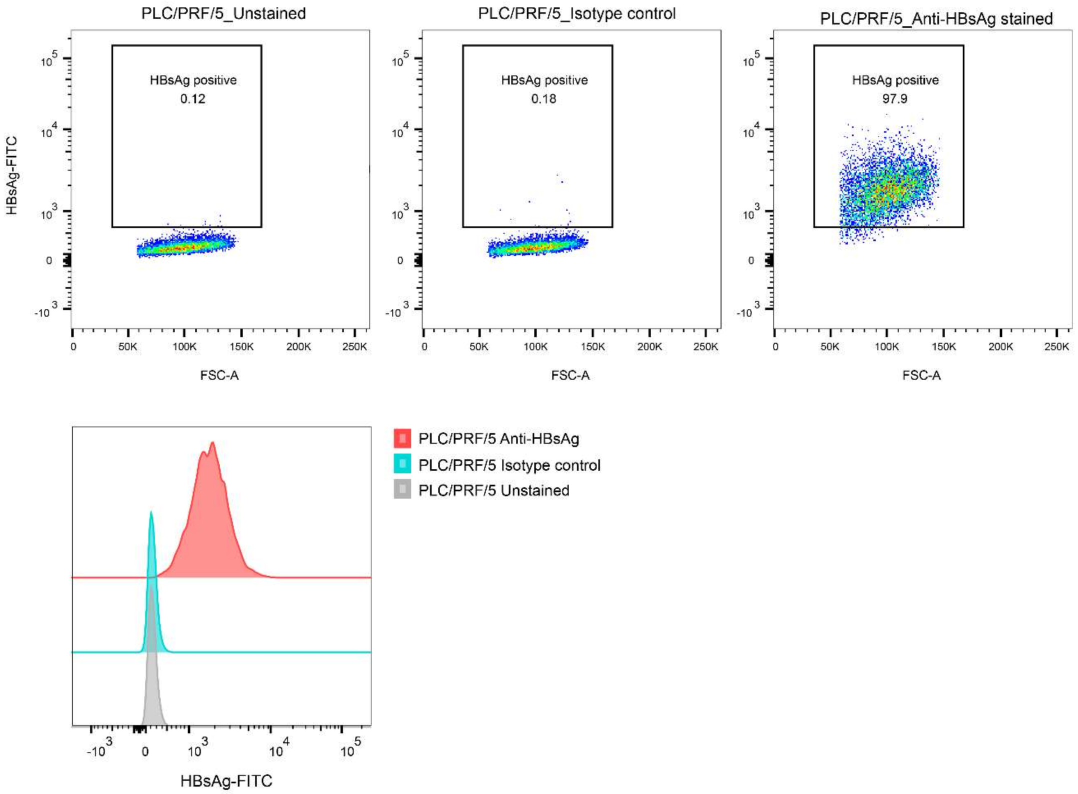


**Supplementary Figure 9. HBsAg expression in PLC/PRF/5 cells.** PLC/PRF/5 cells were fixed, permeabilised and stained with FITC conjugated anti-HBsAg antibody. Bivariate density plot and histogram indicate the expression of intracellular HBsAg in PLC/PRF/5 HCC cell line and is representative of two independent experiments.

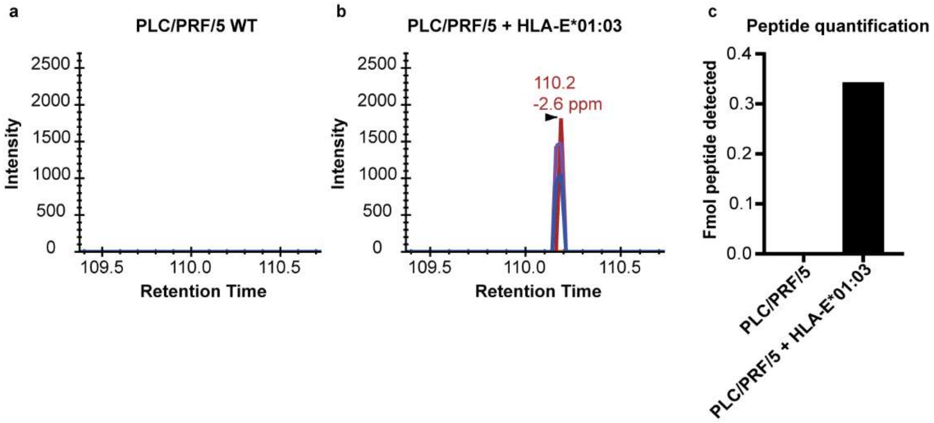


**Supplementary Figure 10. HBV Env$_{371\text{-}379}$ (L6I) peptide identification by mass spectrometry.** Fragment ion intensities obtained when analysing wild type PLC/PRF/5 cells (**a**) or PLC/PRF/5 cells overexpressing HLA-E*01:03 (**b**) by parallel reaction monitoring mass spectrometry. **c** Absolute amount of peptide detected in samples when using a ratio metric approach comparing endogenous peptide levels to the stable heavy isotope labelled internal peptide standard. Source data are provided as a Source Data file.

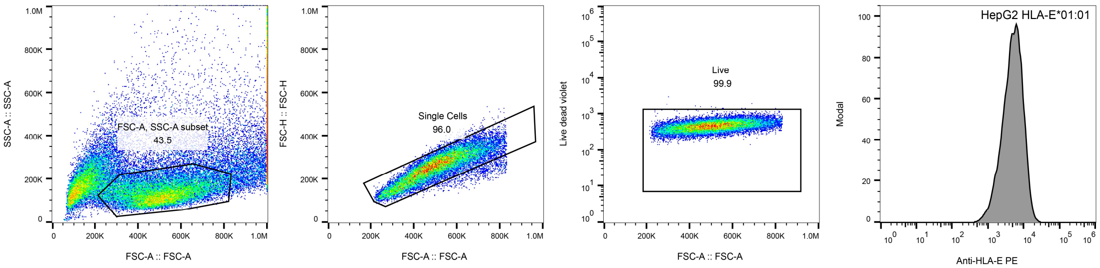


**Supplementary Figure 11.** Gating strategy of HepG2 cells transduced with HLA-E*01:01 or HLA-E*01:03 analysed and presented on Fig. 6a.

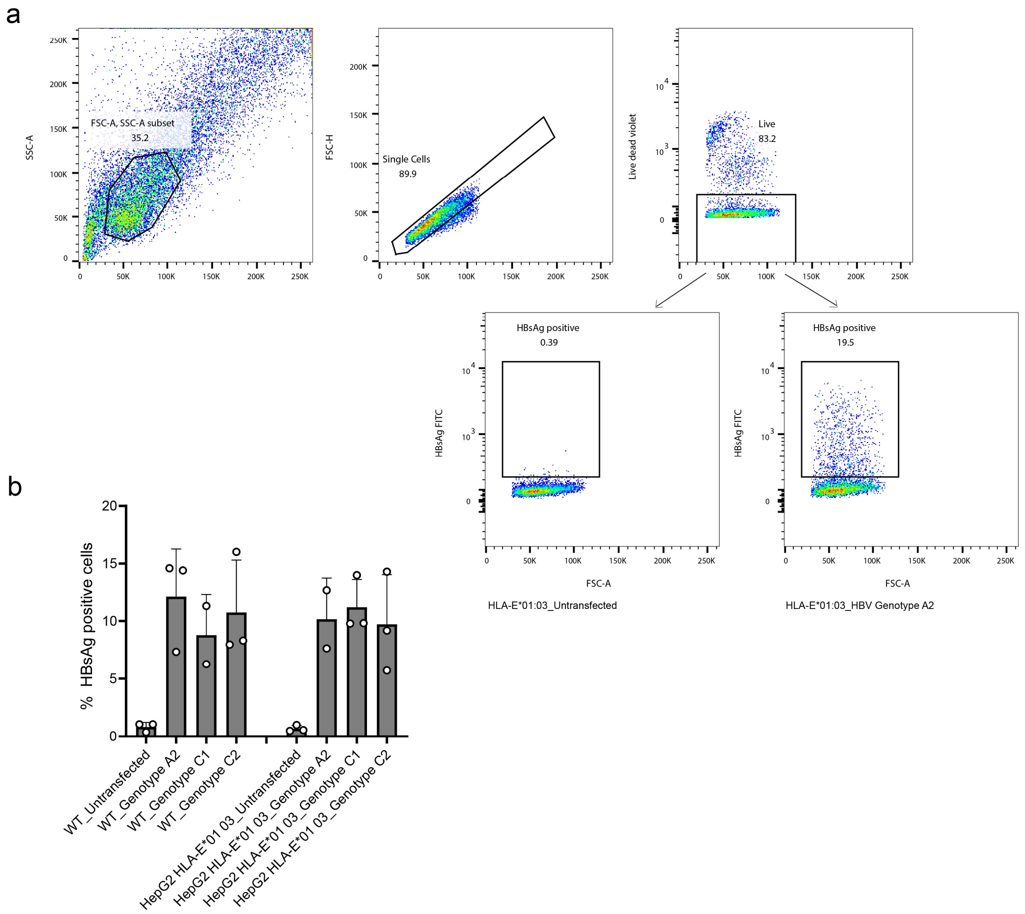


**Supplementary Figure 12. Flow cytometry quantification of HBV infection of HepG2 cells.** Wildtype and HLA-E*01:03 overexpressing HepG2 cells were transfected with HBV plasmids encoding Genotype A2 ($Env_{371-379}$ (L6I)), C1 ($Env_{371-379}$ (S3N)) and C2 ($Env_{371-379}$). 24 h post-transfection cells were fixed, permeabilised and stained with FITC conjugated anti-HBsAg. **a** Gating strategy used to analyse singlets and live HBsAg positive cells. **b** Graph represents percentage of cells expressing intracellular HBsAg. Data are represented as mean ± SD from three independent experiments. Source data are provided as a Source Data file.

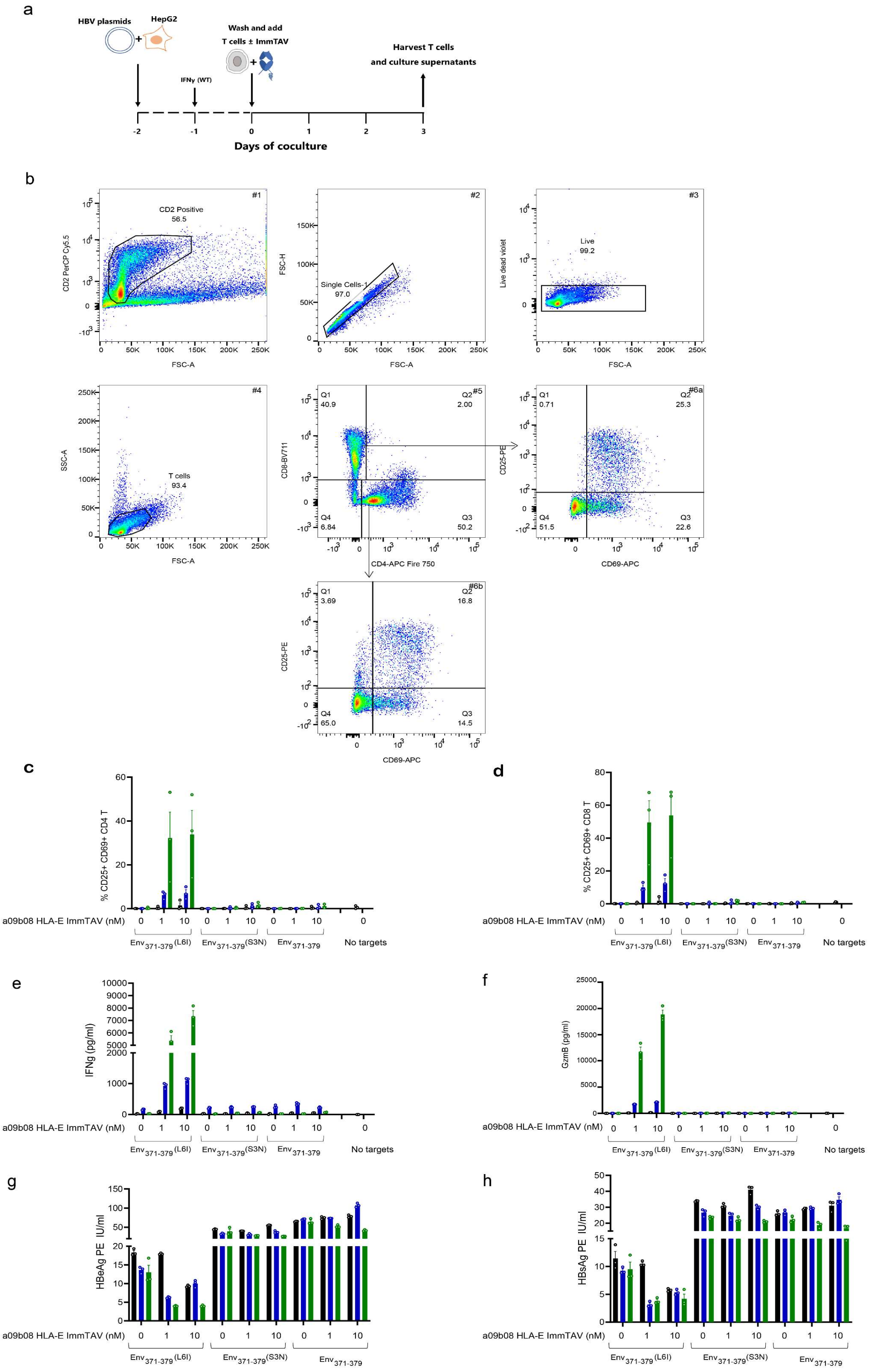
a
HBV plasmids
HepG2
IFNγ (WT)
Wash and add
T cells ± ImmTAV
Harvest T cells
and culture supernatants
-2
-1
0
1
2
3
Days of coculture
b
CD2 Positive
56.5
CD2 PerCP Cy5.5
FSC-A
Single Cells-1
97.0
FSC-H
Live
99.2
Live dead violet
T cells
93.4
SSC-A
CD8-BV711
CD4-APC Fire 750
Q1 40.9
Q2 2.00
Q3 50.2
Q4 6.84
CD25-PE
CD69-APC
Q1 0.71
Q2 25.3
Q3 22.6
Q4 51.5
Q1 3.69
Q2 16.8
Q3 14.5
Q4 65.0
c
% CD25+ CD69+ CD4 T
a09b08 HLA-E ImmTAV (nM)
Env371-379(L6I)
Env371-379(S3N)
Env371-379
No targets
d
% CD25+ CD69+ CD8 T
e
IFNg (pg/ml)
f
GzmB (pg/ml)
g
HBeAg PE IU/ml
h
HBsAg PE IU/ml

**Supplementary Figure 13. The a09b08 ImmTAV activates T cells to eliminate HBV-transfected HepG2 cells. a** HepG2 wildtype and HLA-E*01:03 overexpressing cells were transfected with replication competent HBV plasmids encoding genotype A2 ($Env_{371-379}$ (L6I)), C1 ($Env_{371-379}$ (S3N)) and C2 ($Env_{371-379}$)). 24 h post-transfection, some wells of HepG2 wildtype cells were stimulated with IFN-γ (1 ng/mL) for 16 h. Cells were washed and co-cultured with pan T cells (1:1 ratio) with or without a09b08 ImmTAV at concentrations 1 nM and 10 nM. Pan T cells and culture supernatants were harvested 72 h (end of co-culture) according to the schematics shown (modified from Fergusson et al., 2020[1]). Pan T cells were stained for the activation markers (CD25 and CD69) and analysed by flow cytometry. Panel a is released under a Creative Commons Attribution-Non Commercial 4.0 International license (https://creativecommons.org/licenses/by-nc/4.0/deed.en). **b** Gating strategy used to analyse CD2+ pan T cells, singlets, viable CD4 and CD8 T cells and their activation status. The percentage of $CD4^+$ (**c**) and $CD8^+$ (**d**) T cells expressing CD69 and CD25 at 72 hours were plotted as mean ± SEM of 3 donors. Levels of granzyme B (**e**) and IFN-γ (**f**) in the culture supernatants at 72 hours were quantified using MSD. Data represents the mean ± SEM of triplicates (n=3). HBeAg (**g**) and HBsAg (**h**) levels in the culture supernatant at day 3 were quantified using ELISA. Dots represent values from three independent experiments and error bars represent mean ± SEM (n=7). Source data are provided as a Source Data file.

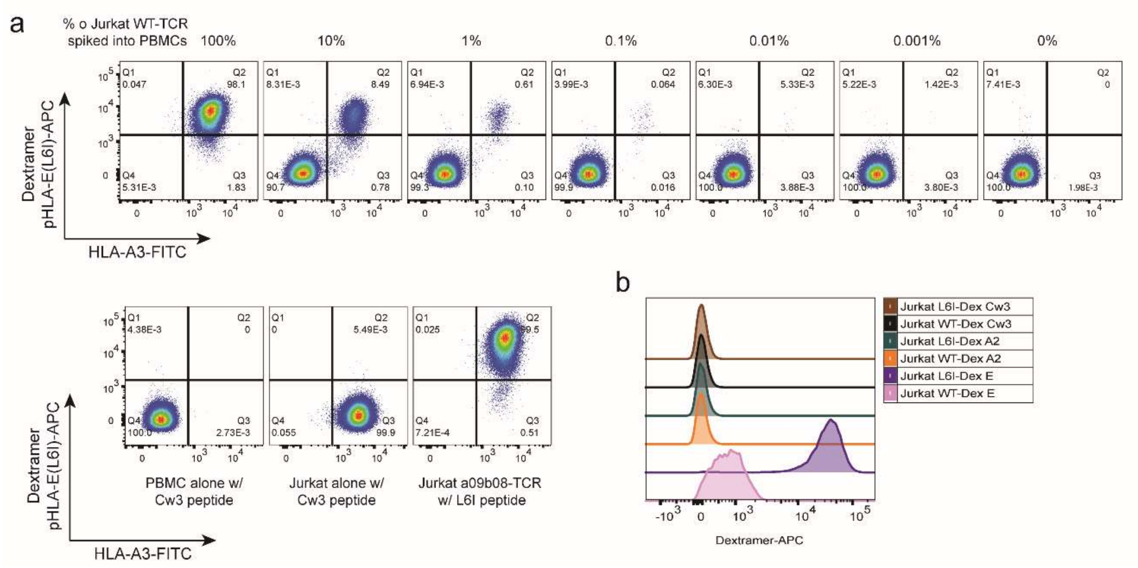


**Supplementary Figure 14. Sensitivity of the dextramer-E reagent to recognize HLA-E restricted HBV-specific CD8+ T cells and TCR Jurkat clones. a** Jurkat clones transduced with wild type TCR were spiked into HBV-naïve PBMC at different concentrations ranging from 100-0.001% and were stained with dextramer-HLA-E $Env_{371-379}$ (L6I) to assess the sensitivity of the reagent. Jurkat cells transfected with high affinity a09b08 TCR were used as positive control. Jurkat cells are HLA-A3 positive. **b** Jurkat clones transduced with wild type (WT) and high affinity a09b08 TCR (L6I) were stained with dextramer-HLA-E $Env_{371-379}$ (L6I) (pink and purple histograms), HLA-A*02:01 $Env_{371-379}$ (L6I) (yellow and dark green histograms), or the HLA-E*01:03 Cw3 signal peptide (black and brown histograms) to test the dextramer specificity.

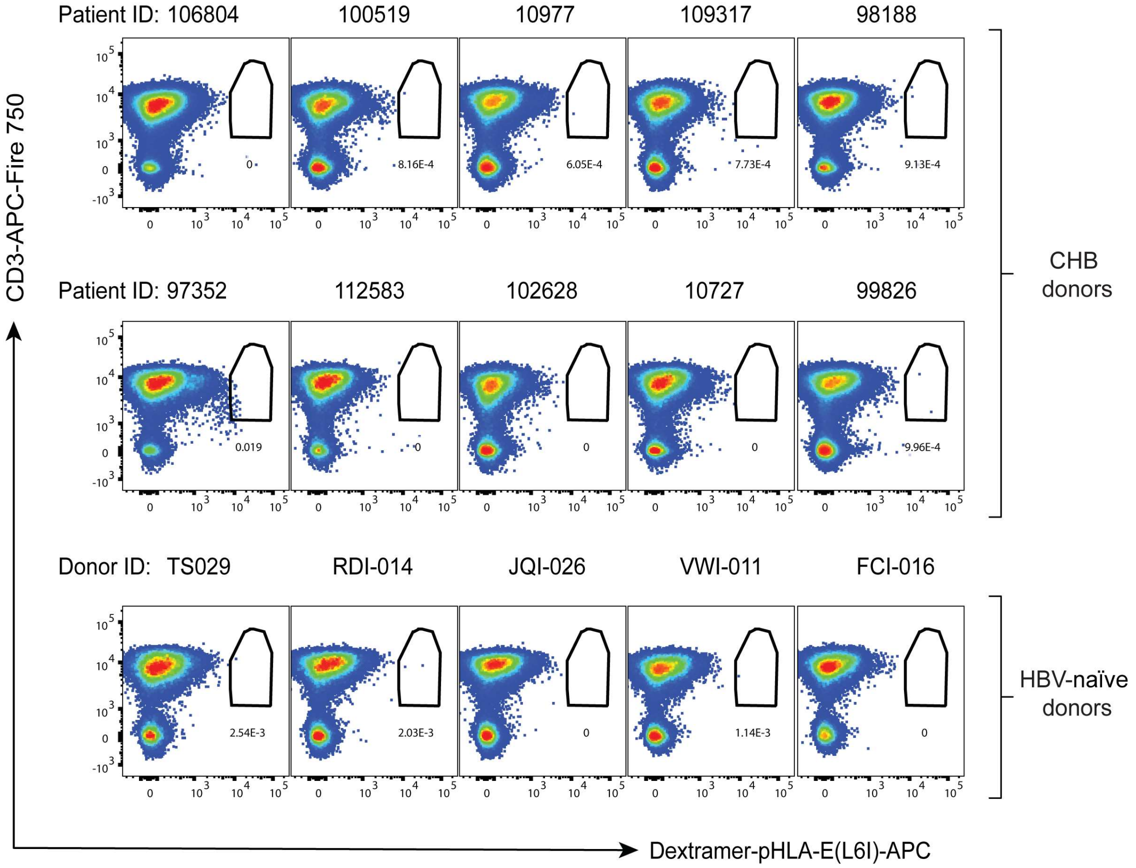


**Supplementary Figure 15. Detection of HBV antigen-specific T cells by pHLA-E multimers from fresh PBMC.** PBMC from CHB donors (n=10) and HBV-naïve donors (n=5) were incubated with dextramer-HLA-E $Env_{371\text{-}379}$ (L6I) to detect % of $CD3^+$ dextramer-$E^+$ cells (dextramer positive cells were gated as live $CD3^+$ singlet cells).

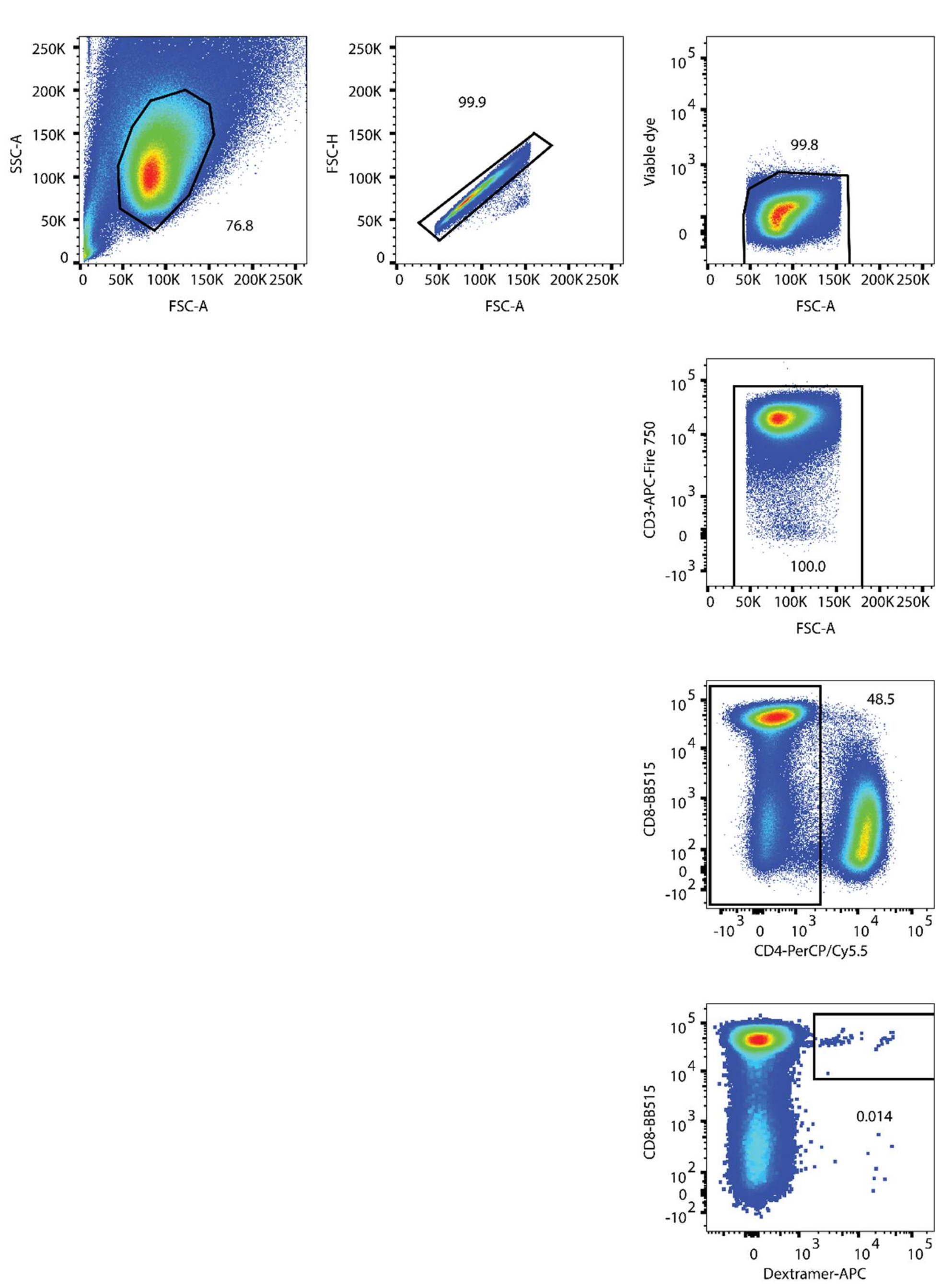


**Supplementary Figure 16. Flow cytometry measurement of HLA-E restricted HBV CD8$^+$ T cell frequency.** Gating strategy used to analyse the singlet, viable cell population, CD3$^+$ population, CD8$^+$ population and dextramer-E positive CD8$^+$ T cell population in HBV-naïve and CHB donor samples.

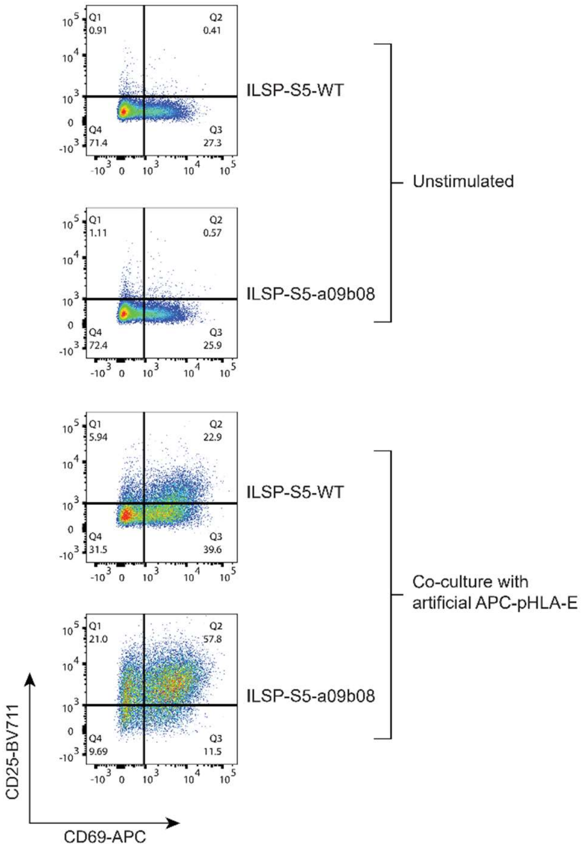


**Supplementary Figure 17. Artificial antigen presenting cell (aAPC) activate Jurkat cells expressing wt and a09b08 TCRs after co-culture.** aAPCs conjugated to L6I-pHLA-E and anti-CD28 antibody were co-cultured overnight with Jurkat cells transduced with either wt TCR or a09b08 TCR; cells were analysed by flow cytometry for the upregulation of activation markers CD25 and CD69.

| HBV peptide | Genotype prevalence<br>A<br>(n=586) | <br>B<br>(n=3313) | <br>C<br>(n=1127) | <br>D<br>(n=748) | <br>E<br>(n=235) |
|---|---|---|---|---|---|
| $Env_{371-379}$ | 0.00 | 0.00 | 0.65 | 0.84 | 0.00 |
| $Env_{371-379}$ (S3N) | 0.00 | 0.00 | 0.21 | 0.00 | 0.00 |
| $Env_{371-379}$ (L6I) | 0.45 | 0.16 | 0.09 | 0.03 | 0.91 |

**Supplementary Table 1. Genotype prevalence (A-E) of $Env_{371-379}$, $Env_{371-379}$ (S3N) and $Env_{371-379}$ (L6I) variants worldwide.** Prevalence calculated on the number of full-length sequences analysed per genotype (n).

| | | a09b08 binding (RU) | | |
|---|---|---|---|---|
| pHLA-E*01:03 | pHLA loaded (RU) | Run 1 | Run 2 | Run 3 |
| Leaders A2/Cw3/G | 2090 | -3.3 | -3.2 | -3 |
| Leaders A34/A80/B7/Cw7 | 2097 | -4.8 | -4.7 | -4.5 |
| $Env_{371\text{-}379}$ (L6I) | 687 | 103.4 | 138.9 | 162.8 |

**Supplementary Table 2. The a09b08 ImmTAV does not bind pHLA-E*01:03 complexes loaded with signal peptides.** Summary table of the SPR experimental conditions.

| Molecule | $Env_{371-379}$-HLA-E | $Env_{371-379}$ (S3N)-HLA-E | $Env_{371-379}$ (L6I)-HLA-E |
|---|---|---|---|
| PDB code | 8RLT | 8RLU | 8RLV |
| Space group | $P2_1$ | $P2_1$ | $P2_1$ |
| Unit cell dimensions | a, b, c = 79.70 Å, 155.06 Å, 93.41 Å<br>α, β, γ = 90.00°, 97.04°, 90.00° | a, b, c = 81.42 Å, 147.84 Å, 91.91 Å<br>α, β, γ = 90.00°, 99.03°, 90.00° | a, b, c = 80.18 Å, 153.81 Å, 93.40 Å<br>α, β, γ = 90.00°, 96.63°, 90.00° |
| X-ray source | DLS i04 | DLS i04 | DLS i04 |
| Wavelength (Å) | 0.97950 | 0.97950 | 0.97950 |
| Resolution range (Å) | 79.57-2.25 (2.29-2.25) | 65.51-2.35 (2.39-2.35) | 79.65-2.61 (2.65-2.61) |
| Completeness (%) | 98.6 (95.8) | 99.4 (88.9) | 99.2 (97.3) |
| Multiplicity | 7.2 (7.4) | 6.9 (5.2) | 7.1 (6.8) |
| I/σ I | 10.5 (0.5) | 9.3 (0.6) | 10.7 (0.4) |
| $R_{merge}$ | 0.150 (2.958) | 0.159 (1.606) | 0.142 (3.246) |
| $R_{pim}$ | 0.060 (1.164) | 0.065 (0.758) | 0.057 (1.345) |
| $CC_{1/2}$ | 0.998 (0.493) | 0.992 (0.521) | 0.998 (0.313) |
| Unique reflections | 104999 (5115) | 88665 (3947) | 67783 (3323) |
| Refinement | | | |
| $R_{work}$ / $R_{free}$ (%) | 20.94/25.21 | 20.83/26.09 | 19.76/25.06 |
| RMS (bonds) | 0.008 | 0.008 | 0.008 |
| RMS (angles) | 1.574 | 1.567 | 1.625 |
| Mean B-factor ($Å^2$) | 66.90 | 58.72 | 94.35 |

**Supplementary Table 3.** Data processing and refinement statistics for TCR-pMHC complexes. Values in parentheses refer to the outer resolution shell.

| HLA-E | a09b08 TCR (HBV $Env_{371-379}$) | a09b08 TCR (HBV $Env_{371-379}$ (S3N)) | a09b08 TCR (HBV $Env_{371-379}$ (L6I)) |
|---|---|---|---|
| R62 | H94α, N95α | H94α, N95α | H94α, N95α |
| R65 | H94α, N95α, T96α, G97α, R97β | H94α, N95α, T96α, G97α, R97β | H94α, N95α, T96α, G97α, R97β |
| S66 | H94α | H94α | H94α |
| D69 | Y31β, R97β | Y31β, R97β | Y31β, V50β, R97β |
| Q72 | E30β, Y31β V50β, S51β | E30β, S51β | E30β, V50β, S51β |
| I73 | | E30β | E30β |
| R75 | S51β | E30β | E30β, S51β |
| V76 | E30β | E30β | E30β |
| A150 | R95β | R95β | R95β |
| E152 | | | R95β |
| E154 | Y51α, K56α | Y51α, K56α | Y51α, L52α, K56α |
| H155 | Y51α, T99β, E100β | Y51α, T99β | Y51α, T99β, E100β |
| R157 | L52α | L52α | L52α |
| A158 | Y51α, L52α | Y51α, L52α | Y51α, L52α |
| E161 | L52α | L52α | L52α |
| D162 | N53α, K67α | K67α | N53α, K67α |
| | | | |
| Peptide | | | |
| I1 | H94α | H94α | H94α |
| P4 | H94α | H94α | H94α |
| F5 | V31α, Y51α, N96β | V31α, Y51α, N96β | V31α, Y51α, N96β |
| L6 (I6) | N96β | N96β | N96β |
| L8 | R95β | R95β | R95β |

**Supplementary Table 4. List of TCR-pHLA-E interactions for the three complexes determined using a distance cut-off of 4 Å.**

| Experimental conditions tested (Figure 4a) | | | $EC_{50}$ (pM ImmTAV) | | | |
|---|---|---|---|---|---|---|
| ImmTAV | Target cell | Target peptide | Donor 1 | Donor 2 | Donor 3 | Average |
| Titration | THP-1-E*01:03 | 10 µg/mL L6I | 3.97 | 5.32 | 1.79 | 3.69±1.5 |
| | THP-1-E*01:01 | | 14.3 | 8.13 | 20.3 | 14.2±5.0 |
| | THP-1-E*01:03 | 10 µg/mL S3N | 5.34 | 4.79 | 11.8 | 7.31±3.2 |
| | THP-1-E*01:01 | | 127 | 104 | 224 | 152±52 |
| | THP-1-E*01:03 | 10 µg/mL index | 60.9 | 73.7 | 224 | 120±74 |
| | THP-1-E*01:01 | | ND | ND | ND | ND |
| Experimental conditions tested (Figure 4b) | | | $EC_{50}$ (µg/mL peptide) | | | |
| ImmTAV | Target cell | Target peptide | Donor 1 | Donor 2 | Donor 3 | Average |
| 1 nM | THP-1-E*01:03 | Titration of L6I | 1.43E-03 | 4.45E-04 | 1.25E-03 | 1.04E-03±4.3E-04 |
| | THP-1-E*01:01 | | 1.15 | 0.485 | 0.447 | 0.695±0.3 |
| | THP-1-E*01:03 | Titration of S3N | 0.35 | 0.171 | 0.137 | 0.219±0.1 |
| | THP-1-E*01:01 | | 4.02 | 4.31 | 6.36 | 4.9±1.0 |
| | THP-1-E*01:03 | Titration of index | 9.89 | 4.90 | 11.4 | 8.74±2.8 |
| | THP-1-E*01:01 | | ND | ND | ND | ND |
| Experimental conditions tested (Figure 4c) | | | $EC_{50}$ (pM ImmTAV) | | | |
| ImmTAV | Target cell | Target peptide | Donor 1 | Donor 2 | Donor 3 | Average |
| Titration | HepG2 minigene | L6I minigene | 4.44 | 2.38 | 4.15 | 3.66±0.9 |
| | | S3N minigene | 342 | 107 | ND | 224±117.5 |
| | | Index minigene | ND | ND | ND | ND |

**Supplementary Table 5.** $EC_{50}$ (pM or µg/mL) values, averages, and standard deviations for all PBMC donors discussed in Fig. 4. For each individual donor the average of triplicate measurements is shown.

| Cell line | HLA A alleles | HLA B alleles | HLA C alleles | HLA-E alleles | Equivalent HLA leader peptides* |
|---|---|---|---|---|---|
| HEPG2 | A*02:01<br>A*24:02 | B*35:14<br>B*51:08 | C*04:01<br>C*16:02 | E*01:01<br>E*01:01 | A2, B15, Cw3, Cw16 |
| PLCPRF5 | A*03:01<br>A*33:01 | B*42:02<br>B*53:01 | C*04:01<br>C*17:01 | E*01:03<br>E*01:03 | A1, B7, B15, Cw3, Cw7 |
| THP-1 | A*02:01<br>A*02:01 | B*15:11<br>B*15:11 | C*03:03<br>C*03:03 | E*01:03<br>E*01:03 | A2, B15, Cw3 |

**Supplementary Table 6.** MHC Class I alleles and leader peptides of cell lines. HLA-E genotyping was performed in house as described in materials and methods. *Leader peptide sequences as described in Supplementary Table 7.

| Peptide name | Abbreviated peptide name | Description | Peptide Sequence | HLA-E*01:01 INF-γ release (SFU ± SD) | HLA-E*01:03 INF-γ release (SFU ± SD) |
|---|---|---|---|---|---|
| HBV Env$_{371-379}$ | Index | Env$_{371-379}$ peptide | ILSPFLPLL | ND | ND |
| HBV Env$_{371-379}$ (L6I) | L6I | Env$_{371-379}$ (L6I) variant | ILSPFIPLL | 1406±77.5 | 1386±45.9 |
| HBV Env$_{371-379}$ (S3N) | S3N | Env$_{371-379}$ (S3N) variant | ILNPFLPLL | ND | ND |
| Leader A1 | LA1 | HLA leader sequence peptides[2] | VMAPRTLLL | 67.8±14.9 | 65.6±28.4 |
| Leader A2 | LA2 | | VMAPRTLVL | 50.5±6.8 | 64.5±9.8 |
| Leader A34 | LA34 | | IMAPRTLVL | 55.5±6.8 | 54.5±18.9 |
| Leader A80 | LA80 | | VMPPRTLLL | 44.8±8.6 | 61.1±14.3 |
| Leader B7 | LB7 | | VMAPRTVLL | 43.3±7.8 | 48.7±7.3 |
| Leader B13 | LB13 | | VTAPRTLLL | 53±13.4 | 56±17.9 |
| Leader B15 | LB15 | | VTAPRTVLL | 42.8±13.4 | 40±17.4 |
| Leader Cw3 | LCw3 | | VMAPRTLIL | 51.6±26.3 | 53.8±30.0 |
| Leader Cw7 | LCw7 | | VMAPRALLL | 30.3±12.9 | 44.1±22.1 |
| Leader Cw16 | LCw16 | | VMAPQALLL | 33.3±16.4 | 49.1±19.2 |
| Leader G | LG | | VMAPRTLFL | 44.8±19.8 | 48.1±19.7 |
| Mimetic peptide 1 | Mimetic peptide 1 | Potential mimetic peptides from the human proteome* | ALSWRLPLL | 61.6±21.8 | 54.1±15.6 |
| Mimetic peptide 2 | Mimetic peptide 2 | | ILQRFLPLI | 60.5±20.8 | 48.6±29.1 |
| Hsp60 signal peptide | Hspd1$_{10-18}$ | Self HLA-E binding peptide[3] | QMRPVSRVL | 43.8±6.36 | 41.8±9.9 |
| Mtb RLPA | Mtb RLPA | Mtb HLA-E binding peptides[4,5] | RLPAKAPLL | 67.1±12.6 | 72.1±12.8 |
| MTB MmmpL8$_{75-83}$ | MTB mmpL8$_{75-83}$ | | ILPSDAPVL | 52.6±17.39 | 54.8±17.4 |
| HCV Core$_{36-44}$ | HCV Core$_{36-44}$ | Hepatitis C virus HLA-E binding peptide[5,6] | LLPRRGPRL | 61.1±21.8 | 60±30.1 |
| ImmTAV + PBMCs | | | | 16.2±13.4 | 8.3±6.1 |
| ImmTAV+PBMC+Targets (no peptide) | | | | 48.2±8.2 | 77.8±36.5 |

**Supplementary Table 7. Details of peptides used for ELISpot and/or peptide pulsing assays.** Average IFN-γ responses from 2 HBV-naïve donor PBMC in the presence of 1 nM ImmTAV and THP-1-E cells pulsed with 10 μg/mL of either Env$_{371-379}$ (L6I) peptide or other indicated HLA-E known binding peptides. *Human 9-mer peptides identified from the human proteome with similar properties to the target peptide. SFU = average number of spot forming units. Error bars mean ± S.D (standard deviation) of two PBMC donors each tested in triplicate. Source data are provided as a Source Data file.

| Patient ID | Age | Gender | HBV viral load (IU/mL) | HBsAg detection | HLA typing | | | Variants identified | |
|---|---|---|---|---|---|---|---|---|---|
| | | | | | HLA-A alleles | HLA-B alleles | HLA-C alleles | Major Variant | Minor Variant |
| 106804 | 36 | Female | 171 | Positive | A*33:03:01:01<br>A*74:01:01:01 | B*15:03:01<br>B*15:10:01 | C*02:10:01:01<br>C*03:04:02:01 | L6I<br>(ILSPFIPL*) | L6I<br>(ILSPFIPLL) |
| 100519 | 50 | Female | 175 | Positive | A*11:01:01:01<br>A*29:02:01:01 | B*15:02:01<br>B*44:03:01 | C*08:01:01<br>C*16:01:01 | L6I<br>(ILSPFIPLL) | L6M<br>(ILSPFMPL(L/*) |
| 10977 | 48 | Male | Undetected | Nonreactive | A*01:01:01:01<br>A*24:02:01:01 | B*08:01:01<br>B*15:01:01 | C*04:01:01<br>C*07:01:01 | L6I<br>(ILSPFIPLL) | ND |
| 109317 | 43 | Female | 9670300 | Positive | A*24:02:01:01<br>A*30:01:01:01 | B*40:02:01<br>B*46:01:01 | C*01:02:01<br>C*03:03:01 | Index<br>(ILSPFLPLL) | L8V<br>(ILSPFLPVL) |
| 98188 | 49 | Female | <20 | Positive | A*02:01:01:01<br>A*29:02:01:01 | B*35:01:01<br>B*57:01:01 | C*04:01:01<br>C*07:01:01 | Index<br>(ILSPFLPLL) | Several minor peaks |
| 97352 | 46 | Female | Undetected | Nonreactive | A*30:01:01:01<br>A*30:02:01:01 | B*13:02:01<br>B*18:01:01 | C*05:01:01<br>C*06:02:01 | Index<br>(ILSPFLPLL) | ND |
| 112583 | 44 | Female | <20 | Nonreactive | A*03:02:01:01<br>A*33:01:01:01 | B*14:02:01<br>B*27:05:02 | C*02:02:02<br>C*08:02:01 | L6I<br>(ILSPFIPLL) | Index<br>(ILSPFLPLL) |
| 102628 | 36 | Female | 312 | Positive | A*32:01:01:01<br>A*33:03:01:01 | B*07:06:01<br>B*56:01:01 | C*01:02:01<br>C*08:02:01 | L2VL6I<br>(IVSPFIPLL) | Several minor peaks |
| 10727 | 49 | Female | 2191 | Positive | A*02:01:01:01<br>A*29:01:01:01 | B*07:02:01<br>B*51:02:01 | C*07:02:01<br>C*15:02:01 | I1TL6M<br>(TLSPFMPLL) | L6M<br>(ILSPFMPLL) |
| 99826 | 60 | Female | <20 | Positive | A*01:01:01:01<br>A*68:02:01:01 | B*07:02:01<br>B*15:10:01 | C*03:04:02<br>C*07:02:01 | S3N<br>(ILNPFLPLL) | ND |

**Supplementary Table 8: Clinical characteristics, HLA class I haplotype and Env target sequence of patients with chronic HBV infection.** HBV DNA (IU/mL) levels and HBsAg detection in the blood were provided by Sanguine Bioscience. HBV viral DNA was isolated from serum and amplified by PCR to sequence the surface antigen region. Both the major and minor variant sequences detected by NGS sequencing are mentioned. *Stop codon. ND = not detected.

| Antibody | Clone | Company | Catalog number | Dilution |
|---|---|---|---|---|
| CD2-PerCP Cy5.5 | RPA-2.10 | Biolegend | 300216 | 01:50 |
| CD4-APC/Cy7 | SK3 | Biolegend | 344616 | 01:50 |
| CD8 BV711 | RPA-T8 | Biolegend | 301043 | 01:50 |
| CD69-APC | FN50 | Biolegend | 310910 | 01:50 |
| CD25-PE | M-A251 | Biolegend | 302606 | 01:50 |
| CD3-APC-Fire 750 | SK7 | Biolegend | 344840 | 01:50 |
| CD4-PerCP/Cy5.5 | RPA-T4 | Biolegend | 300530 | 01:50 |
| CD8-BB515 | RPA-T8 | Biolegend | 564526 | 01:50 |
| FITC anti-HBsAg | polyclonal | Abcam | ab21021 | 01:25 |
| HLA-E-PE | 3D12 | Biolegend | 342604 | 01:100 |
| CD25-BV711 | M-A251 | Biolegend | 356138 | 01:50 |
| HLA-A3-FITC | GAP.A3 | Invitrogen | 11-5754-42 | 01:50 |
| PE Mouse IgG1, κ Isotype | MOPC-21 | BD Pharmingen | 559320 | 1:50 |
| HLA-E | 3D12 | Invivo Biotech services | N/A | 10 μg/mL |
| HLA-A2 | BB7.2 | Invivo Biotech services | N/A | 10 μg/mL |

**Supplementary Table 9. List of antibodies used in this study.** HLA-E and HLA-A2 monoclonal antibodies were ordered and manufactured for Immunocore Ltd. N/A = not applicable. Antibodies validation was done by the manufacturer.

| Cell line | Supplier | Catalogue number | Media | Modifications |
|---|---|---|---|---|
| K562 | ATCC* | CCL-243 | R10 | Lentiviral transduction *with $\beta_2$m-HLA-E*01:01* or *$\beta_2$m-HLA-E*01:03* single chain gene-fusion constructs |
| HEPG2 | ATCC* | HB 8065 | E10 | Stable transfection with minigene encoding peptide of interest (ILSP index, L6I, or S3N). Lentiviral transduction with full length *β2m-HLA-E*01:03*. |
| PLC/PRF/5 | Public Health England | 85061113 | D10 | Lentiviral transduction with full length *$\beta_2$m-HLA-E*01:03* |
| THP-1 | ATCC* | TIB202 | R10 | CRISPR-Cas9 knockout of *B2M and CIITA*; lentiviral transduction with *$\beta_2$m-HLA-E*01:01* or *$\beta_2$m-HLA-E*01:03* single chain gene-fusion constructs |

**Supplementary Table 10 Cell lines, suppliers, and culture conditions.** R10 = RPMI media supplemented with 1% (v/v) penicillin/streptomycin, 2 mM L-glutamine, and 10% fetal calf serum (FCS); D10 and E10 are DMEM and EMEM supplemented as R10. *ATCC = American Type Culture Collection (Manassas, USA).

| Primer Name | Primer direction | Primer sequence 5’-3’ | Application |
|---|---|---|---|
| HLA-E-genotyping_Fwd | Forward | GGTCTCACACCCTGCAGTGGA | HLA-E genotyping |
| HLA-E-genotyping_Rev | Reverse | AGCCCTGTGGACCCTCTT | HLA-E genotyping |
| $\beta_2$m-sgRNA_Fwd | Forward | CTCGCGCTACTCTCTCTTTC | CRISPR-KO |
| $\beta_2$m-sgRNA_Rev | Reverse | GGCCACGGAGCGAGACATCT | CRISPR-KO |
| CIITA-sgRNA_Fwd | Forward | CTACCACTTCTATGACCAGA | CRISPR-KO |
| CIITA-sgRNA_Rev | Reverse | CATCGCTGTTAAGAAGCTCC | CRISPR-KO |
| HBV surface Ag_Fwd | Forward | GACTYGTGGTGGACTTCTC | PCR |
| HBV surface Ag_Rev | Reverse | TCAGCAAAYACTYGGCA | PCR |
| HBV nested_Fwd | Forward | TGGATGTGTCTGCGGCGTTTTATCAT | PCR |
| HBV nested_Rev | Reverse | ATDCKTTGACANACTTTCCAATCAA | PCR |
| HBV-sequencing_Fwd | Forward | CACHTGTATTCCCATCCCA | Sanger Sequencing |

**Supplementary Table 11. List of primers used in this study.**

## Supplementary References

1. Fergusson, J. R. *et al.* Immune-Mobilizing Monoclonal T Cell Receptors Mediate Specific and Rapid Elimination of Hepatitis B–Infected Cells. *Hepatology* **72**, 1528–1540 (2020).

2. Pietra, G. *et al.* HLA-E-restricted recognition of cytomegalovirus-derived peptides by human CD8+ cytolytic T lymphocytes. *Proc. Natl. Acad. Sci.* **100**, 10896–10901 (2003).

3. Michaëlsson, J. *et al.* A Signal Peptide Derived from hsp60 Binds HLA-E and Interferes with CD94/NKG2A Recognition. *J. Exp. Med.* **196**, 1403–1414 (2002).

4. Joosten, S. A. *et al.* Mycobacterium tuberculosis Peptides Presented by HLA-E Molecules Are Targets for Human CD8+ T-Cells with Cytotoxic as well as Regulatory Activity. *Plos Pathog* **6**, e1000782 (2010).

5. Barber, C. *et al.* Structure-guided stabilization of pathogen-derived peptide-HLA-E complexes using non-natural amino acids conserves native TCR recognition. *Eur J Immunol* (2022) doi:10.1002/eji.202149745.

6. Molero-Abraham, M., Lafuente, E. M., Flower, D. R. & Reche, P. A. Selection of Conserved Epitopes from Hepatitis C Virus for Pan-Populational Stimulation of T-Cell Responses. *J. Immunol. Res.* **2013**, 601943 (2013).
