# Supplementary material for: Viral sequence determines HLA-E-restricted T cell recognition of hepatitis B surface antigen
Source: Nat Commun. 2024 Nov 22;15:10126. doi: 10.1038/s41467-024-54378-9 (PMC11584656; doi:10.1038/s41467-024-54378-9)
Supplement: Supplementary file 2 — Description of Additional Supplementary Files [file 41467_2024_54378_MOESM2_ESM.pdf]

Description of Additional Supplementary Files

Supplementary Data 1: List of peptides (9 and 10 mer) derived from HBV Env proteins based on sequence conservation across the five main HBV genotypes (A, B, C, D, E) and on netMHCpan4.0 affinity prediction, synthesized and screened for their ability to bind to recombinant human HLA-E*01:03 using a thermal shift assay. L – large envelope protein; M – middle envelope protein; S – small envelope protein. [a] <2% = weak binding, <0.5% = strong binding. [b]ND = not able to determine Tm (non-binding peptide). Error bars mean ± S.D. (standard deviation).

Supplementary Data 2: Estimated percentage of CHB patients' coverage of $Env_{371\text{-}379}$, $Env_{371\text{-}379}$ (S3N) and $Env_{371\text{-}379}$ (L6I) variants based on the extrapolation of genotypes A-E distribution worldwide (Velkov et al., 2018).
