# Supplementary material for: Viral sequence determines HLA-E-restricted T cell recognition of hepatitis B surface antigen
Source: Nat Commun. 2024 Nov 22;15:10126. doi: 10.1038/s41467-024-54378-9 (PMC11584656; doi:10.1038/s41467-024-54378-9)
Supplement: Supplementary file 5 — Reporting Summary [file 41467_2024_54378_MOESM5_ESM.pdf]

nature portfolio

Corresponding author(s): Luis F. Godinho

Last updated by author(s): Oct 16, 2024

Reporting Summary

Nature Portfolio wishes to improve the reproducibility of the work that we publish. This form provides structure for consistency and transparency in reporting. For further information on Nature Portfolio policies, see our Editorial Policies and the Editorial Policy Checklist.

Statistics

For all statistical analyses, confirm that the following items are present in the figure legend, table legend, main text, or Methods section.

n/a	Confirmed	
☐	☒	The exact sample size (*n*) for each experimental group/condition, given as a discrete number and unit of measurement
☐	☒	A statement on whether measurements were taken from distinct samples or whether the same sample was measured repeatedly
☐	☒	The statistical test(s) used AND whether they are one- or two-sided<br>*Only common tests should be described solely by name; describe more complex techniques in the Methods section.*
☒	☐	A description of all covariates tested
☒	☐	A description of any assumptions or corrections, such as tests of normality and adjustment for multiple comparisons
☐	☒	A full description of the statistical parameters including central tendency (e.g. means) or other basic estimates (e.g. regression coefficient) AND variation (e.g. standard deviation) or associated estimates of uncertainty (e.g. confidence intervals)
☐	☒	For null hypothesis testing, the test statistic (e.g. *F*, *t*, *r*) with confidence intervals, effect sizes, degrees of freedom and *P* value noted<br>*Give P values as exact values whenever suitable.*
☒	☐	For Bayesian analysis, information on the choice of priors and Markov chain Monte Carlo settings
☒	☐	For hierarchical and complex designs, identification of the appropriate level for tests and full reporting of outcomes
☒	☐	Estimates of effect sizes (e.g. Cohen's *d*, Pearson's *r*), indicating how they were calculated

Our web collection on statistics for biologists contains articles on many of the points above.

Software and code

Policy information about availability of computer code

Data collection	MUSCLE - multiple alignments of protein sequences; Quantstudio 6; BD FACSDiva v6.1.2; Sony Biotechnology v2.1.5. BIAcoreTM T200; BIAcoreTM 8K
Data analysis	NetMHCpan-4.0 - peptide-MHC class I interactions predictions integrating eluted ligand and peptide binding affinity data; BLOSUM62 - matrix developed to analyze the frequency of amino acids substitutions in clusters of related proteins. GraphPad Prism 10 - for statistical analysis. FlowJo_v10.7.1 - for flow cytometry analysis. Biacore Insight Evaluation 3.0.12.15655 - for binding kinetics. Quantstudio 6 - for thermal shifts analysis. Pymol 2.5.7 - for structural models analysis.

For manuscripts utilizing custom algorithms or software that are central to the research but not yet described in published literature, software must be made available to editors and reviewers. We strongly encourage code deposition in a community repository (e.g. GitHub). See the Nature Portfolio guidelines for submitting code & software for further information.

Data

Policy information about availability of data

All manuscripts must include a data availability statement. This statement should provide the following information, where applicable:

- Accession codes, unique identifiers, or web links for publicly available datasets
- A description of any restrictions on data availability
- For clinical datasets or third party data, please ensure that the statement adheres to our policy

The crystallography data generated in this study have been deposited in the RCSB protein data bank (PDB) with the accession codes 8RLT [https://www.rcsb.org/

structure/8RLT], 8RLU [https://www.rcsb.org/structure/8RLU] and 8RLV [https://www.rcsb.org/structure/8RLV]. All data are included in the Supplementary Information or available from the authors, as are unique reagents used in this Article. The raw numbers for charts and graphs are available in the Source Data file whenever possible. Scripts for data processing and analysis are publicly available at https://github.com/Immunocore/FindPeptideOrthologs.

Research involving human participants, their data, or biological material

Policy information about studies with human participants or human data. See also policy information about sex, gender (identity/presentation), and sexual orientation and race, ethnicity and racism.

Reporting on sex and gender	The authors are aware of the sex (male or female) from the HBV patient samples obtained from Sanguine Biosciences. The authors are not aware of the sex from anonymized healthy volunteers.
Reporting on race, ethnicity, or other socially relevant groupings	The authors are not aware of the race, ethnicity, or other socially relevant groupings from healthy or patient samples.
Population characteristics	The donor cohort provided by Sanguine Biosciences, is made up of nine women and one man and the average age is 46 years (36-60 years).
Recruitment	10 subjects diagnosed with HBV were recruited by Sanguine Biosciences®. PBMC and serum samples were sourced from Sanguine Biosciences®. The selection criteria for the HBV patients: be in the age group of 18-85 years; be diagnosed with hepatitis B; and having detectable levels of HBV DNA in the blood and/or been off HBV treatment for at least three months. Any subjects who have taken any investigational product within last 30 days of sample collection and with concurrent infection with other Hepatitis viruses or HIV were excluded from the study. All the patient samples were collected after proper consent and according to ethical guidelines.
Ethics oversight	Anonymized healthy volunteers who consented to donate at Immunocore as part of a UK Health Research Authority approved study. HBV patient volunteers had to sign a consent form (SAN-BB-01) provided by Sanguine Biosciences and a copy of the California Experiments Subject's Bills of Right. The study protocol (REC reference 13/SC/0226) was approved by the Oxford A Research Ethics Committee.

Note that full information on the approval of the study protocol must also be provided in the manuscript.

Field-specific reporting

Please select the one below that is the best fit for your research. If you are not sure, read the appropriate sections before making your selection.

☒ Life sciences ☐ Behavioural & social sciences ☐ Ecological, evolutionary & environmental sciences

For a reference copy of the document with all sections, see nature.com/documents/nr-reporting-summary-flat.pdf

Life sciences study design

All studies must disclose on these points even when the disclosure is negative.

Sample size	For most in vitro experiments using human PBMCs, at least three donors were tested. For the detection of HLA-E restricted CD8 T cells specific for the HBV target, 10 HBV-positive donors and 5 healthy donors were tested.
Data exclusions	No data were excluded
Replication	Experiments were repeated independently or performed with technical replicates. The number of repeats is provided in the figure legend
Randomization	The experiments were not randomized
Blinding	Investigators were not blinded during experiments or result analysis.

Reporting for specific materials, systems and methods

We require information from authors about some types of materials, experimental systems and methods used in many studies. Here, indicate whether each material, system or method listed is relevant to your study. If you are not sure if a list item applies to your research, read the appropriate section before selecting a response.

Materials & experimental systems

n/a	Involved in the study	
[ ]	[x]	Antibodies
[ ]	[x]	Eukaryotic cell lines
[x]	[ ]	Palaeontology and archaeology
[x]	[ ]	Animals and other organisms
[x]	[ ]	Clinical data
[x]	[ ]	Dual use research of concern
[x]	[ ]	Plants

Methods

n/a	Involved in the study	
[x]	[ ]	ChIP-seq
[ ]	[x]	Flow cytometry
[x]	[ ]	MRI-based neuroimaging

Antibodies

Antibodies used

Antibodies used for flow cytometry (from Biolegend unless annotated):
CD2-PerCP Cy5.5 (clone: RPA-2.10, Catalog #: 300216, 1:50 dilution)
CD4-APC/Cy7 (clone: SK3, Catalog #: 344616, 1:50 dilution)
CD8 BV711 (clone: RPA-T8, Catalog #: 301043, 1:50 dilution)
CD69-APC (clone: FN50, Catalog #: 310910, 1:50 dilution)
CD25-PE (clone: M-A251, Catalog #: 302606, 1:50 dilution)
CD3-APC-Fire 750 (clone: SK7, Catalog #: 344840, 1:50 dilution)
CD4-PerCP/Cy5.5 (clone: RPA-T4, Catalog #: 300530, 1:50 dilution)
CD8-BB515 (clone: RPA-T8, Catalog #: 564526, from BD Biosciences, 1:50 dilution)
FITC anti-HBsAg (clone: polyclonal, Catalog #:ab21021, from Abcam; 1:25 dilution)
HLA-E-PE (clone:3D12, Catalog #:342604, 1:100 dilution)
CD25-BV711 (clone: M-A251, Catalog #:356138, 1:50 dilution)
HLA-A3-FITC (clone: GAP.A3, Catalog #:11-5754-42, 1:50 dilution)
PE Mouse IgG1, κ Isotype (clone: MOPC-21, Catalog #:559320, from BD Pharmingen,1:50 dilution)

Monoclonal antibodies used for blocking experiments were ordered and manufactured by Invivo Biotech Services, Germany.
HLA-E (clone: 3D12, 10 μg/mL)
HLA-A2 (clone: BB7.2, 10 μg/mL)

Validation

For all the commercially available antibodies validation was done by manufacturer. For flow cytometry, antibodies were used as suggested by the commercial vendors and determined by experimental optimization.

Eukaryotic cell lines

Policy information about cell lines and Sex and Gender in Research

Cell line source(s)

K562 supplier ATCC catalogue number CCL-243; HEPG2 supplier ATCC catalogue number HB 8065; PLC/PRF/5 supplier Public Health England catalogue number 85061113; THP-1 supplier ATCC catalogue number TIB202

Authentication

All cell lines were authenticated by the supplier. Short tandem repeat (STR) testing to authenticate the cell lines was also performed in-house to validate cell lines.

Mycoplasma contamination

All cell lines tested negative for mycoplasma contamination

Commonly misidentified lines
(See ICLAC register)

No commonly misidentified cell lines were used

Plants

Seed stocks

Report on the source of all seed stocks or other plant material used. If applicable, state the seed stock centre and catalogue number. If plant specimens were collected from the field, describe the collection location, date and sampling procedures.

Novel plant genotypes

Describe the methods by which all novel plant genotypes were produced. This includes those generated by transgenic approaches, gene editing, chemical/radiation-based mutagenesis and hybridization. For transgenic lines, describe the transformation method, the number of independent lines analyzed and the generation upon which experiments were performed. For gene-edited lines, describe the editor used, the endogenous sequence targeted for editing, the targeting guide RNA sequence (if applicable) and how the editor was applied.

Authentication

Describe any authentication procedures for each seed stock used or novel genotype generated. Describe any experiments used to assess the effect of a mutation and, where applicable, how potential secondary effects (e.g. second site T-DNA insertions, mosiacism, off-target gene editing) were examined.

Flow Cytometry

Plots

Confirm that:

- [x] The axis labels state the marker and fluorochrome used (e.g. CD4-FITC).
- [x] The axis scales are clearly visible. Include numbers along axes only for bottom left plot of group (a 'group' is an analysis of identical markers).
- [x] All plots are contour plots with outliers or pseudocolor plots.
- [x] A numerical value for number of cells or percentage (with statistics) is provided.

Methodology

Sample preparation	Adherent cells were harvested by trypsinisation and suspension cells were transfered by pipetting, labeled with the previously described antibodies, and analyzed. Live/Dead fixable violet dye for flow cytometry (Invitrogen; L34955, 01:1000)
Instrument	BD LSR Fortessa X-20 or Sony SH800S
Software	For data collection: BD FACSDiva v6.1.2 or Sony Biotechnology v2.1.5<br>For data analysis: FlowJo_v10.7.1
Cell population abundance	Flow cytometry analysis was performed with purified Pan T cell population which are >95% pure to define antigen specific cells which are about 0.01%
Gating strategy	Singlets were gated using FSC-H/FSC-A; Fixable viable dye/FSC-A defined the living cell population. The gating for each specific cell population is described in the gating strategy in the supplemental data.

- [x] Tick this box to confirm that a figure exemplifying the gating strategy is provided in the Supplementary Information.
